# A systematic review on intra-arterial cerebral infusions of chemotherapeutics in the treatment of glioblastoma multiforme: The state-of-the-art

**DOI:** 10.3389/fonc.2022.950167

**Published:** 2022-09-23

**Authors:** Mateusz Pinkiewicz, Milosz Pinkiewicz, Jerzy Walecki, Michał Zawadzki

**Affiliations:** ^1^ Department of Diagnostic Imaging, Mazowiecki Regional Hospital in Siedlce, Siedlce, Poland; ^2^ English Division, Faculty of Medicine, Wroclaw Medical University, Wroclaw, Poland; ^3^ Division of Interventional Neuroradiology of the Central Clinical Hospital of the Ministry of Interior and Administration, Department of Radiology, Centre of Postgraduate Medical Education, Warsaw, Poland

**Keywords:** glioblastoma, IA chemotherapy, SIACI, glioblastoma chemotherapy, IA delivery, bevacizumab in glioblastoma, cetuximab in glioblastoma

## Abstract

**Objective:**

To provide a comprehensive review of intra-arterial cerebral infusions of chemotherapeutics in glioblastoma multiforme treatment and discuss potential research aims. We describe technical aspects of the intra-arterial delivery, methods of blood-brain barrier disruption, the role of intraoperative imaging and clinical trials involving intra-arterial cerebral infusions of chemotherapeutics in the treatment of glioblastoma multiforme.

**Method:**

159 articles in English were reviewed and used as the foundation for this paper. The Medline/Pubmed, Cochrane databases, Google Scholar, Scielo and PEDro databases have been used to select the most relevant and influential papers on the intra-arterial cerebral infusions of chemotherapeutics in the treatment of glioblastoma multiforme. Additionally, we have included some relevant clinical trials involving intra-arterial delivery of chemotherapeutics to other than GBM brain tumours.

**Conclusion:**

Considering that conventional treatments for glioblastoma multiforme fall short of providing a significant therapeutic benefit, with a majority of patients relapsing, the neuro-oncological community has considered intra-arterial administration of chemotherapeutics as an alternative to oral or intravenous administration. Numerous studies have proven the safety of IA delivery of chemotherapy and its ability to ensure higher drug concentrations in targeted areas, simultaneously limiting systemic toxicity. *Nonetheless*, the scarcity of phase III trials prevents any declaration of a therapeutic benefit. Given that the likelihood of a single therapeutic agent which will be effective for the treatment of glioblastoma multiforme is extremely low, it is paramount to establish an adequate multimodal therapy which will have a synergistic effect on the diverse pathogenesis of GBM. Precise quantitative and spatial monitoring is necessary to guarantee the accurate delivery of the therapeutic to the tumour. New and comprehensive pharmacokinetic models, a more elaborate understanding of glioblastoma biology and effective methods of diminishing treatment-related neurotoxicity are paramount for intra-arterial cerebral infusion of chemotherapeutics to become a mainstay treatment for glioblastoma multiforme. Additional use of other imaging methods like MRI guidance during the procedure could have an edge over X-ray alone and aid in selecting proper arteries as well as infusion parameters of chemotherapeutics making the procedure safer and more effective.

## Introduction

Glioblastoma (GBM) multiforme is the most common type of brain cancer, accounting for approximately 40% of all primary malignant brain tumours (1–3).

This distinct pathological entity is known for its aggressive progression and poor prognosis, with a median patient survival duration of 14-17 months in the case of contemporary clinical trials and ~12 months in population-based studies (1–3). Only 5% of patients manage to achieve a 5-year survival. Standard therapy has consisted of surgical resection, external beam radiation or both (4).

Since its publication in 2005, the Stupp protocol, consisting of radiotherapy (2 Gy per/day x 30 days, 60 Gy total) and oral temozolomide (75 mg/m2), has been the gold standard for the treatment of glioblastoma multiforme (GBM) (5, 6). Nonetheless, the majority of patients relapse after six months (7). Consequently, despite the concerted efforts of the medical community, available methods of treatment fall short of providing any significant improvements, causing GBM to be incurable. The failure of conventional chemotherapy to increase overall survival is attributable to low penetration of the blood-brain barrier and systemic toxicity (5–8). Consequently, aside from intra-arterial delivery, researchers have been driven to explore other drug delivery methods, such as intrathecal, intracavitary and convection-enhanced delivery. However, although preclinical studies demonstrated promising results, these novel approaches to drug delivery require further clinical investigation before they become the mainstay of treatment (8).

Intra-arterial chemotherapy of GBM is not a new concept, in fact, it is one of the oldest treatments attempted for this deadly disease, introduced in the 50s coincidently with the introduction of radiotherapy for brain tumours (9). The underlying hypothesis behind intra-arterial drug administration was that achieving a higher concentration of the pharmaceutic in the specified area of the tumour would lead to an increased likelihood of tumour cell death. Furthermore, the possibility of reducing the toxicity, so pronounced in the case of the systematic approach, could also provide the opportunity of using higher doses of chemotherapeutics (10). These potential advantages resulted in a considerable body of literature reporting the use of IA delivery in the 50s, 70s and 90s. Nonetheless, the significant neurotoxicity of chemotherapeutics available at the time eventually discouraged further research (10, 11).

As of now, approximately 3000 IA dd procedures have been reported all over the world (12). This is attributable to the growth of personalised oncology, improvement of imaging techniques, and new endovascular tools. Developments like dual lumen balloons, large-bore distal access catheters, and soft microcatheters allow for modification of blood flow in the brain vessels to an unprecedented degree. Intra-arterial infusions do not require craniotomy, are easy to repeat, and in experienced hands are safe and reproducible. Nonetheless, although the intra-arterial route seems to be the most physiological way to administer any drug to the brain, there is a substantial obstacle in overcoming the blood-brain barrier, responsible for blocking the majority of drugs from entering the brain tissue (13). Although mannitol remains the widely-used method for transient BBB disruption, there are numerous promising techniques being developed. Almost all published intra-arterial infusions were performed under X-ray guidance in cath labs designed to treat pathologies of relatively big vessels in the brain. X-ray angiography has a high spatial resolution, accurately depicts the intracranial vessels, and allows for safe microcatheter navigation into distal intracranial arteries. However, the possibility to visualise parenchymal flow in brain tumours and surrounding tissue is limited. Real-time monitoring of infusion has recently become possible in MRI during the procedure. First such procedures were already performed in humans. Moreover, combining interventional MR with PET can even further expand the ability to monitor chemical processes and labelled-drug accumulation in the brain in a real-time manner.

A wide range of new therapeutics administered intra-arterially may not only include chemotherapeutics but also antibodies, cells (e.g. carTcells), modified viruses or radiotherapeutics. Before we start composing new trials, we should thoroughly know why our predecessors failed. Some anecdotal, spectacular successes will also be analysed and gathered in this review.

## Methodological approach

### Search strategy and selection criteria

A systematic literature review was carried out to review all available relevant data. During the article selection process, the authors followed the recommendations made by the Preferred Reporting Items for Systematic Reviews and Meta-Analyses (PRISMA). All authors independently have searched the Medline/Pubmed, Cochrane databases, Google Scholar, Scielo and PEDro databases by using the following keywords “Glioblastoma”, “IA chemotherapy”, “SIACI”, “SSIACI”, “glioblastoma treatment”, “glioblastoma chemotherapy”, “IA delivery”, “bevacizumab in glioblastoma”, “cetuximab in glioblastoma”.

Additional search has included the Scielo and PEDro databases. The last search was conducted in May 2022. The references of the publications of interest were also screened for relevant papers.

### Study selection and data extraction

All of the selected articles were read in full text. Only papers written in English have been considered. Non-peer-reviewed papers and records not available in the full text have not been included. Also, studies were excluded if there was incomplete or missing data. We have excluded conference abstracts. The eligibility and quality of publications have been independently evaluated by three reviewers. We have chosen articles for inclusion on the grounds of study quality and design. The primary selection had no limitations regarding the publication date. We have included studies focusing on technical aspects of IA delivery, established and new methods of blood-brain barrier disruption, drugs used for intra-arterial cerebral infusions for the treatment of glioblastoma multiforme and intraoperative imaging. Additionally, we have described novel studies concerning gene and cell therapy. We have reviewed and included selected preclinical and clinical studies concerning IA therapy for glioblastoma multiforme. Some papers describing emerging therapies for glioblastoma multiforme have also been reviewed and added. The judgments concerning the risk of bias were formed by a single reviewer and subsequently double-checked by another reviewer

### Results

**Figure 1:**
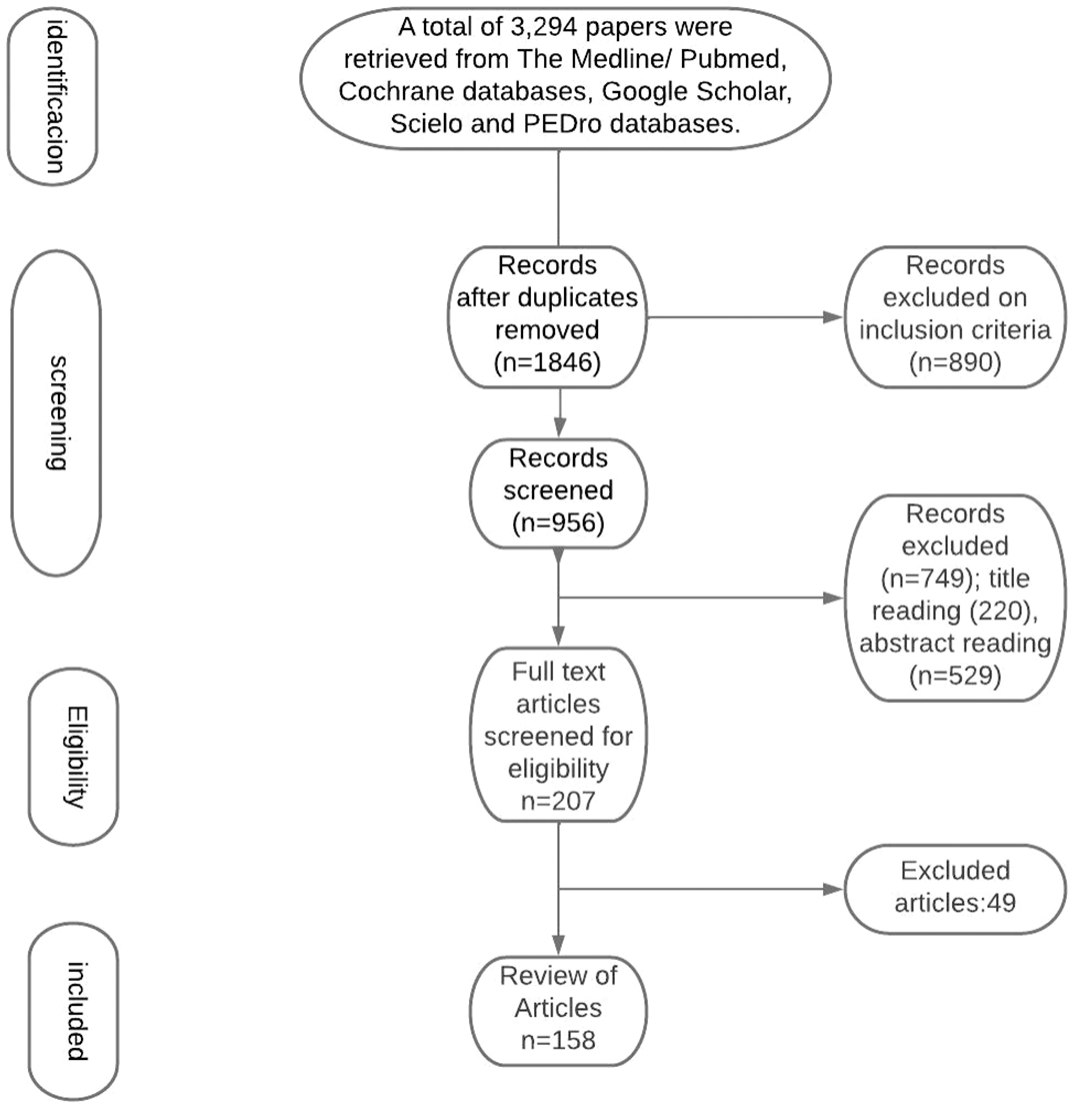
Flow Diagram represents our process of article selection.

A total of 3,294 papers were retrieved from The Medline/Pubmed, Cochrane databases, Google Scholar, Scielo and PEDro databases. Screening for duplicates and their removal resulted in a total of 1846 articles. Subsequently, we have excluded 890 articles due to language and study design. Titles or abstracts of 1068 articles were screened, obtaining 207 papers not meeting any exclusion criterion. After full-text evaluation, we have excluded 48 papers. This has led to the inclusion of 159 articles. The flow diagram represents our process of article selection (**Figure 1**).

### Historical aspect

Intra-arterial delivery of chemotherapeutics has been pioneered by Bierman et al. (14) and Klopp et al. (15) who have designed techniques for the delivery of high-doses of nitrogen mustard directly to the liver tumours *via* its arterial blood supply (10). Multiple administrations of nitrogen mustard responsible for tumour regression in rabbits with extracranial xenografts have prompted Klopp et al., as well as inspired French et al., to use IA delivery of chemotherapeutics in humans for malignant progressive gliomas (15, 16). However, despite the enthusiasm, nitrogen mustard delivery was associated with poor therapeutic benefits and significant damage to the hematopoietic system (15). In the 1970s, Eckmann had lent credence to previous assumptions made by Wilson et al. and successfully proved that IA delivery of chemotherapeutics allowed obtaining higher drug concentrations in targeted tumours than that in non-targeted tissues (17–19). Stanley Rapaport’s findings concerning the fundamental role of tight junctions in BBB permeability, as well as the demonstration that hyperosmolar BBB disruption causes dehydration of endothelial cells and subsequent disruption of tight junctions in a reversible fashion, have laid the groundwork for Neuwelt research which proved that hyperosmolar BBB disruption increased concentrations of chemotherapies in targeted sites for central nervous system lymphomas (20–25). In 1978, Levin et al. reported that IA infusion offered a 2.5–5-fold increase in drug delivery drug over IV infusion after comparing intravenous to intracarotid artery (ICA) administration of 14C-labelled carmustine in squirrel monkeys (26). Multiple studies following have broadened the substantive scaffolding, further highlighting the efficacy of the intra-arterial delivery of chemotherapeutics into the vessels supplying the brain (10, 21–25). Given that the chemotherapeutics available at the time were associated with significant neurotoxicity, the interest in IA delivery slowly began to fade (10, 11, 25–27). This, paradoxically, has started to take place at the height of technological advances in endovascular methods (10, 11). Although numerous preclinical and clinical studies have proven the validity of that is intra-arterial delivery of chemotherapeutics, not until the development of new drugs and availability of new selective microcatheters and other endovascular devices did the interest of the IA once again awaken.

### Blood-brain barrier disruption

The blood-brain barrier, responsible for the cellular and molecular exchange between the blood vessels and brain parenchyma, is highly selective, blocking the passage of ionised molecules whose molecular weights are larger than 180 Da (11, 21, 28, 29). Given that most chemotherapeutics’ size is in the range of 200-1200 Da, the blood-brain barrier is a significant impediment in the treatment of brain tumours (28). Even if some drugs manage to penetrate the BBB, they usually fail to reach effective local concentrations (11). The poor prognosis of GBM is in large part due to the lack of successful drug delivery through the blood-brain tumour barrier (BBTB). The BBTB consists of already existing and newly formed blood vessels, which are responsible for the delivery of nutrients and oxygen to the tumour, as well as the glioma cell migration to other parts of the brain (29). High expression of VEGF and subsequent angiogenesis result in the formation of abnormal vessels and a flawed BBTB (29). Although the BBTB of high-grade gliomas is considered to be “leaky”, lower-grade gliomas have an almost fully functional BBB, prompting the passage of chemotherapeutics to be ineffective (29). Glioblastomas are known to have different regions of BBTB integrity. Bulky tumours are characterised by completely dysfunctional BBTB, less invasive circumferential regions have a leaking BBTB, whereas areas far from the tumour bulk can display a fully functional BBTB (29). Overexpression of receptors that mediate ligand-dependent drug delivery has been observed in brain tumour capillaries. This could be exploited to selectively enhance drug delivery to tumour tissues (29, 30). Extensive research over the last decades has resulted in various methods of brain-barrier disruption. This section will focus on describing available methods as well as discuss the nearest future.

#### Osmotic blood-brain barrier disruption

Rapoport et al. were the first to demonstrate that an intra-arterial infusion of a hyperosmotic solution of mannitol will result in a temporary shrinkage of endothelial cells due to the creation of an osmotic gradient and, consequently lead to the opening of the tight junctions (20, 22). Reportedly, osmotic disruption can increase the levels of successfully infused chemotherapeutics by up to 90-fold (11). Non-selective opening of the BBB results in an increase of brain fluid, as well as in an influx of molecular compounds, which can lead to neurological toxicity, aphasia and hemiparesis (29, 31). Thus, using hypertonic solutions demands caution. Nonetheless, studies have shown that osmotic disruption can be safe and of therapeutic value in capable hands (31–37). Due to individual factors, there is no exact dose and infusion time. Numerous research has described osmotic BBB disruption by intra-arterial infusion of 1.37 mmol/L mannitol (25%) (38–40). Boockvar et al. report relying on the mannitol infusion rate of 0.083 mL/s for 120 seconds, whereas Siegal et al. infused at a rate of 3 to 11 ml/second over 30 seconds (10, 31). Despite the staggering amount of more than 4200 osmotic BBB disruption procedures having been performed at multiple centres in more than 400 patients, there is no consensus in regard to the maximum permeability effect of osmotic BBB disruption (31). According to Siegal et al., the maximum effect in humans lasts up to 40 minutes which is preceded by a rapid decline in permeability, with the normal threshold restored between 6 and 8 hours after the osmotic disruption (31). These findings differ from those of Zünkeler et al. who used rubidium-82 to measure blood-to-tissue influx and estimated that the mean half time for the return of permeability to almost baseline values was only 8 minutes in the normal brain (31, 36).

#### Bradykinin receptor-mediated BBTB opening

Bradykinin is a potent vasodilator, capable of increasing capillary permeability (41). In 1986, Raymond et al. demonstrated that high doses of bradykinin will result in the breakdown of the normal blood-brain barrier (41). Inamura et al. have successfully proved that low doses of bradykinin led to the selective increase of the permeability of abnormal brain tissue capillaries to low and high molecular weight neuropharmaceuticals (42). This has brought about the clinical development of bradykinin analogs such as Cereport or labramidil (29). Research has demonstrated that using synthetic bradykinin analogs to improve the delivery of IA carboplatin is a safe method, allowing for a two-fold increase in drug delivery. Nonetheless, there was no clear clinical benefit demonstrated in the randomised, double-blind, placebo-controlled phase II study of RMP-7 in combination with carboplatin or in the Phase II trial of intravenous lobradimil and carboplatin used in the treatment of childhood brain tumours (29, 43, 44). This may be due to an inadequate dose level of RMP-7 of 300 ng/kg. However, there were no subsequent studies with different doses, leaving this matter unsettled (29, 43, 44)

#### Magnetic-resonance- guided focused ultrasound

Magnetic-resonance- guided focused ultrasound (MRgFUS) is a promising technology used for the treatment of a variety of neurological disorders. Most importantly, MRgFUS is also used for the opening of the blood-brain barrier (BBB) in combination with intravenously administered microbubbles (45–50). Regional contrast extravasation on the MR images correlates with the amount of delivered drug, thus allowing for precise targeting of BBB disruption (47). According to research, pulsed ultrasound is capable of safely opening the BBB and providing spatial and temporal specificity (45–50). Ultrasound parameters like intensity and sonication time, as well as the size and concentration of intravenously administered microbubbles, decide on the exact extent of BBB opening (45–50). Reportedly, the possible BBB opening is temporary and lasts for almost 4-6 hours after the treatment (47, 50). Consequently, the transport of various chemotherapeutics used for the treatment of brain tumours can be significantly improved (10, 11).

#### Inhibition of drug efflux transporters

Studies have shown that some drugs have an improved brain penetration once drug efflux transporters are absent (51). Therefore, inhibition of such multidrug resistance efflux transporters by specific inhibitors could be an effective method of boosting drug penetration into the brain without altering the integrity of the endothelial layer and tight junctions, which could avoid the potential toxicity observed with BBBD. Pharmaceutical companies aimed to reverse the multidrug resistance phenotype of tumours by developing elacridar and tariquidar, which inhibit ABCB1 and ABCG2. However, given that clinical trials in solid tumours demonstrated failure, the interest in developing inhibitors has waned (52). Nonetheless, the idea of incorporating these reversal agents to enhance BBB drug penetration is wholly different from using these agents to block multidrug resistance in genomically unstable cancer cells. The goal here would be to increase the accessibility of a sanctuary site by targeting ABC transporters in genomically stable endothelial cells. The ability to block drug efflux transporters will strongly depend on finding a potent inhibitor with proper systemic bioavailability and a ‘commuter’ agent with moderate affinity for these efflux transporters.

Pardridge et al. have reported various receptor-mediated uptake systems for improving the brain uptake of drugs and radiopharmaceuticals (53). GRN1005 (formerly ANG1005) is a conjugate of paclitaxel and the angiopep-2 peptide that targets the lipoprotein receptor-related protein 1 (LRP1) and crosses the BBB by transcytosis (54). A Phase I study has demonstrated promising results, which should be further evaluated by ongoing 3 phase II clinical trials for glioma (Clintrials.gov: NCT01967810) and breast cancer brain metastasis (NCT02048059 and NCT01480583). A clinical trial has also shown similarly encouraging outcomes with the use of 2B3-101 (Clintrial.gov ID: NCT01386580, NCT01818713), which is a PEGylated liposome that is conjugated with glutathione (GSH) (55).

One study has demonstrated that the docosahexaenoic transporter Mfsd2a acts by suppressing transcytosis in CNS endothelial cells (56). According to van Tellingen et al., by interfering with its function or expression it could be possible to enhance transcytosis and consequently enhance drug delivery *via* this route (29).

### Novel methods with potential

Laser interstitial thermal therapy (LITT) is an emerging method of delivering targeted thermal therapy and has been used in brain tumour ablations. Research suggests that hyperthermia induced by LITT may result in the disruption of BBB (57). Research on mice has shown that laser ablation is capable of increasing BBB/BTB permeability, with peak permeability occurring within 1 week and lasting up to 30 days after ablation. Furthermore, molecules as large as human IgG (approximately 150 kDa) were able to pass the BBB after LITT (57). Leuthardt et al. have reported increased serum levels of brain-specific enolase, which is limited to the CNS, after laser ablation in patients suffering from recurrent glioblastoma (58). Authors have suggested that increased permeability in the peritumoral region is attributable to LITT and reaches its peak 1-2 weeks from ablation and returns to the normal threshold by 4-6 weeks (58). The obtained time window provides the potential for the enhancement of IA drug delivery (58). Besides the therapeutic benefit, LITT could also be associated with crucial immunological consequences, given that immunoproteins are being continuously released outside the CNS compartment and could trigger an immune response (58). All of these factors prompt LITT to be a highly interesting phenomenon, albeit requiring much more research.

The advancements in nanotechnology could result in using nanoparticles in intra-arterial administration. Nanoparticles (NP) could be modified to cross the BBB through different transport mechanisms and stay at the targeted area for a longer time, allowing for a gradual release of loaded chemotherapy (59–61). Studies have demonstrated the ability of NP to enhance the half-life of the drug in circulation (59–61). According to Zhao et al., the half-life of TMZ was increased to 13.4 h in comparison to 1.8 h of the free drug by encapsulation in a chitosan-based nanoparticle (61). Ongoing clinical trials involving nanoparticle-based cancer treatment in GBM should evaluate their efficacy, thus allowing for further development in NP-treatment.

Convection enhanced delivery relies on the direct and continuous injection of a chemotherapeutic agent under positive pressure by using stereotactically placed intraparenchymal microcatheters, which allow the passage of molecules of different charges and sizes to any part of the brain (62–65). Although showing potential, neuro-oncological clinical trials with CED have demonstrated poor drug distribution to more peripheral areas of diffuse gliomas and drug reflux, leading to complications and subtherapeutic drug concentrations within the tumour target cells (66, 67). Also, CED has more disadvantages, such as the lack of visualisation of the distribution of the infused drug and unacceptable device-related adverse events (68). Ongoing clinical and preclinical imaging studies may optimise drug distribution *via* CED.

Lately, research has shown that by establishing a local positive pressure gradient convection-enhanced delivery (CED) using catheters stereotactically inserted into brain tumours is capable of improving drug delivery into these tumours and surrounding brain tissue (69, 70). Although a Phase I clinical trial evaluating CED of carboplatin has offered a therapeutic benefit for glioblastomas patients, there are numerous side effects resulting from the use of CED, involving headache, seizure, fever, nausea, vomiting, fatigue, erythema, and in some cases liver enzyme perturbations and haematological changes, which are associated with the time and location of the treatment (71–73). More research is required to provide unequivocal evidence for a benefit of CED in glioblastoma patients.

A study has found that TTFields improve membrane permeability by increasing both the number and the size of pores in the membrane of glioma cells (74, 75). Moreover, the authors reported a substantial increase in the uptake of membrane-associating reagents with a size of 20 kDa and no larger than 50 kDa into glioma cells with TTFields that was reversible, returning to normal within 24 of ceasing TTFields treatment (74, 75). Another suggested that by transiently disrupting the localisation of tight-junction proteins such as claudin 5 and ZO-1, the TTFields therapy can interfere with the integrity of the blood-brain barrier (76).

Mannitol continues to be the most effective method for transient BBB disruption. Studies have demonstrated its safety and good tolerance in combination with intra-arterial chemotherapy. Nonetheless, mannitol mediated BBB disruption may cause an unexpected increase in transcapillary transport of anticancer drugs into healthy brain tissues (77). High-frequency and high-amplitude electroencephalography (EEG) signals suggest that an intra-arterial injection of mannitol through the anterior circulation could have a direct effect on the motor cortex, regardless of the chemotherapy regimen or the size and location of the tumour (78). There are numerous complications that could result from mannitol mediated BBB disruption, such as transient aphasia, hemiparesis, or even oedema-induced intracranial herniation (79). However, studies most often report tachycardia, increased intracranial pressure, vomiting, nausea and headache (79).

### Technical aspects of contemporary intra-arterial drug delivery

**Figure 2:**
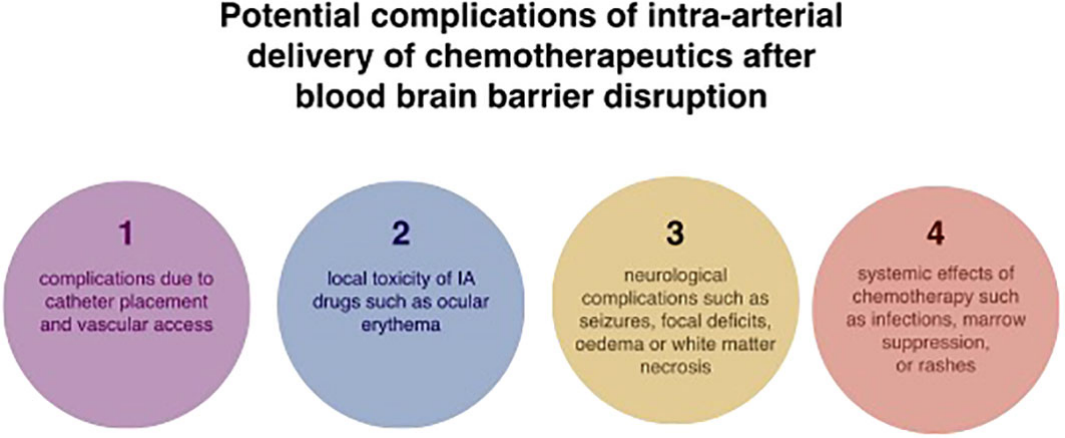
Potential complications associated with intra-arterial drug delivery of chemotherapeutics in the treatment of glioblastoma.

The central concept behind intra-arterial drug administrations was to achieve a higher concentration of the pharmaceutic in the specified area of the tumour and, at the same time, reduce systemic side effects. A Randomised Phase III study comparing intravenous and intra-arterial administrations in newly diagnosed primary glioblastomas patients has shown that intra-arterial delivery of chemotherapeutics has the advantages of smaller toxicity, longer total drug exposure and a higher peak concentration (80). IA injection allows increasing local concentrations of chemotherapeutics to brain tumours up to over 300 times more than the intravenous approach (81). Another study relying on positron emission tomography (PET) measurements has shown that IA delivery had a 50-fold increase in brain tumour tissue concentrations in comparison to IV injections (82). Thanks to the osmotic opening of the blood-brain barrier, IA delivery provided a 300 times higher local concentration of chemotherapeutics to brain tumours than the intravenous approach (81). Technological progress has led to the emergence of selective intra-arterial cerebral infusion (SIACI). This is a technique relying on state-of-the-art microcatheters, which are inserted into the femoral artery and subsequently navigated directly to the tumour supplying vessels (10). This method has an edge over unselective IA infusions like vertebral or carotid infusion as the volume of distribution (Vd) is limited to the targeted area and adjacent tissue sharing the vascular supply (11). Consequently, high selectiveness and reduced neurotoxicity are provided. Microcatheter is navigated with the use of guidewire assistance and road-mapping control in the angiographic suite (10). As much as SIACI is a highly advantageous technique, it is not ideal. To reduce neurotoxicity and assure high drug levels in the corresponding brain region, it is paramount to address the problem of inadequate dosing and “streaming”. Gobin et al. have proposed using a spatial dose fractionation algorithm that selects the proper dose on the basis of cerebral vascular territories rather than weight or body surface area (83). This algorithm relies on the vascular perfusion of the vessel and thus may optimise IA drug delivery (83–85). Various studies blame streaming for high neurotoxicity and unsuccessful treatment (11, 83, 84). Streaming occurs when drugs delivered by the IA method are distributed unequally to different areas of the brain and is caused by the layering of blood flow in the arteries. Some layers stream drugs favourably to one or two arterial branches causing accumulation in supplied areas, while other branches of the same artery do not receive drugs at all. This faulty distribution is attributable to the infusion rate being smaller than 20% of the background blood flow (11, 83). Recognition of this phenomenon has resulted in numerous techniques diminishing this effect. Among them, we can distinguish the incorporation of catheters with side ports, pulsatile injections at rates higher than 20% of the background flow rate and injections during the diastole (11, 86). Furthermore, the notion that tumours with low blood flow respond better to chemotherapy resulted in the use of single or double-balloon catheters to isolate proximal and distal arterial flow, thus successfully maximising local delivery and reducing local and regional complications (10, 11). Research done on computational models and in preclinical settings has vividly shown that cerebral hypoperfusion improves local drug delivery by lowering hydrodynamic stress on the injected molecules and increasing drug transit time through cerebral circulation. Consequently, the pure drug is delivered to the vascular endothelium and opsonization by serum proteins and blood cells is significantly decreased (10, 11, 87–90). As with any operative technique, there are associated risks. These include complications resulting from vascular access and subsequent catheter positioning, systemic toxicities associated with chemotherapy, and, most importantly, the possibility of seizures (33, 39). Reportedly, abnormally small carotid arteries or the presence of two branches rather than three or more increases the possibility of neurologic complications (84, 89, 91). Potential complications associated with intra-arterial drug delivery of chemotherapeutics in the treatment of glioblastoma are shown in **Figure 2**.

### Clinical IA drug deliveries in glioblastoma patients

The more profound understanding of glioblastomas multiforme, the technological improvements and promising outcomes associated with IA chemotherapy in the treatment of retinoblastoma (92), breast cancer (93), head and neck tumours (94) and advanced liver cancers (95, 96) have propelled researches to attest the efficacy of IA chemotherapy in the treatment of glioblastoma multiforme. **Table 1** summarises completed clinical studies focusing on treating glioblastoma multiforme and other brain entities with selective or nonselective IA chemotherapeutic delivery (32, 80, 81, 83, 97–115)

**Table 1:** Summary of studies using intra-arterial delivery of chemotherapeutics for the treatment of GBM.

Study (year)	Number of patients	Study type and phase	Brain tumors	Chemotherapeutic agent Dose	Method of delivery	BBBD Agent	Outcome	Neurotoxicity
Doolittle et al. (2000) (97)	221	MC,II	GBM, BSG, AO, O, MET, GCT, PCNSL, PNET	Carboplatin (200 mg/m2)	nS	Mannitol	(79%) achieved SD or better	Stroke (0.93%) and Herniation (1,2%)
Chow et al. (2000) (98)	46	SC,II	RGBM, AO, AAS	Carboplatin (600 mg/hemisphere)	S	Cereport 300 ng/kg	32% SD or better, PFS 2.9 months average, median OS 6.8 months (23/41) and 10.8 months (18/41)	US
Kochi et al. (2001) (99)	42	MC,II†	NGBM	Nimustine (80 mg/m2)	nS	–	Median survival time 17 months/16 months† and PFS 6 months/11 months†	Reversible vision loss (2.4%)
Madajewicz et al. (2000) (100)	83	SC, II†	GBM,AAS	Etoposide (40) mg/m2 and Cisplatin (60 mg/m2)	nS	–	48% PR or better, Median survival time 18 months	Blurred vision (4.8%), Focal seizures (6%)
Ashby and Shapiro (2001) (101)	25	SC, II	RGBM, AAS, AO, AOA	Cisplatin (60 mg/m2)	nS	–	40% SD or better and PFS 4.5 months	Headache, Increased Seizure frequency, and Encephalopathy (45%)
Gobin et al. (2001) (83)	113	SC, not stated	GBM, AAS, MET, other	Carboplatin 100–1400 mg/hemisphere	S	Cereport 300 ng/kg	–	Seizures (7%), Major Neurologic deterioration (<0.6%)
Qureshi et al. (2001) (102)	24	SC, not stated	RGBM,AAS, MET, mixed glioma	Carboplatin 34–277 mg/m2	S	Cereport 147–366 mg/m2	Decreased tumor size in 30%, Median OS > 12 months survival in 12 patients	Transient neurologic deficits (20%), Stroke (4%)
Newton et al. (2002) (103)	25	SC, II	AAS, AOA, AO, O,BSG, ME	Carboplatin (200 mg/m2/d)	nS	–	80% SD or better, PFS 6 months	Transient ischemic attack (8%)
Silvani et al. (2002) (104)	15	SC, II†	NGBM	Carboplatin 200 mg/m2 and ACNU 100 mg/m20	nS	–	78.6%/66% SD, PFS 5.2 months/5.8 months†	Seizures (6.6%), Intracerebral hemorrhage (6.6%)
Fortin et al. (2005) (32)	72	SC, II	GBM,AAS,AO,MET, other	Carboplatin protocol Carboplatin, 400 mg/m2 with Cyclophosphamide, 330–660 mg/m2 IV Etoposide 400mg/m2 IV Methotrexate protocol Methotrexate 5000 mg with cyclophosphamide 500 mg/m2 IV andEtoposide 150 mg/m2 IV	nS	Mannitol	Median survival time 9.1 months, median PFS 4.1 months	Thrombocytopenia; Neutropenia; seizures; orbital myositis
Hall et al. (2006) (105)	8	MC, Retrospective analysis	Recurrent DIPG	Carboplatin, 400 mg/m2 Cyclophosphamide, 660 mg/m2 IV Etoposide 400 mg/m^2^ IV Methotresxate, 5000 mg Cyclophosphamide 1000 mg/m2 IV Etoposide 400 mg/m^2^ IV	nS	Mannitol 25% 4–10 cc/s for 30s	Median PFS 15 months, Median OS 27 months	Thrombocytopenia, Neutropenia, infections, neurological disorientation; hearing loss
Imbesi et al. (2006) (80)	17	SC, III†	NGBM	Nimustin (ACNU) 80–100 mg/m2	nS	Mannitol 18% 250 ml	Median OS 17 months, Time to progression was 6 months in case of i.a. ACNU and 4 months for i.v. ACNU	Stroke (5.6%)
Angelov et al. (2009) (106)	149	MC, analysis	PCNSL	Methotrexate not stated, etoposide (150 mg/m2 IV) or cyclophosphamide (15 mg/kg IV and procarbazine (100 mg orally d. Between 1994 and 2005, etoposide or etoposide phosphate (150 mg/m2 IV days) and cyclophosphamide (500 mg/m2 IV) were used.	nS	Mannitol 25%	Median overall survival was 3.1 years (25% estimated survival at 8.5 years). Median progression-free survival (PFS) was 1.8 years, with 5-year PFS of 31% and 7-year PFS of 25%	focal seizures (9.2%)
Guillaume et al. (2010) (107)	13	SC, 1	AO,AOA	Carboplatin (IA, 200 mg/m2), etoposide phosphate (IV, 200 mg/m2), and melphalan (IA, dose escalation) every 4 weeks, for up to 1 year	nS	Mannitol	77% SD or better, PFS 11 months	Speech impairment (7.7%), Retinopathy (7.7%)
Boockvar et al. (2011) (108)	30	SC, 1	RGBM, AAS, AO	Bevacizumab 2–15 mg/kg	S	Mannitol 25% 1.4M	Naiüve group: 34.7% median tumor volume reduction Exposed group: 15.7% median tumor volume reduction	Seizures (6.6%)
Shin et al. (2012) (109)	3	SC, I/II	RGBM	Bevacizumab 13 mg/kg Temozolomide 83mL (199mg) + 22mL (53mg) Cetuximab 100mg/m2	S	Mannitol 25% 10 mL	Decreased tumor size a 1 month	Good tolerance
Jeon et al. (2012) (110)	18	I,II	RGBM	Bevacizumab 2-15 mg/kg	S	Mannitol 10 mL 25% 1.4 mol/L	SD at 10 months in 11 patients, PR in 5 patients, progression in 1 patient and mixed response in 1 patient	Good tolerance
Fortin et al. (2014) (111)	51	II	RGBM	Carboplatin 400 mg/m2 Melphalan 10mg/m2	nS	–	Median PFS 23 months, Median OS 11 months, CR in 3 patients, PR in 22 patients, SD in 14 patients, progression of tumor in 12 patients	Hematological complication
Chakraborty et al. (2016) (81)	15	I	RGBM	Cetuximab 100, 200, 250 mg/m2	S	Mannitol 20% 12,5 ml/120s	–	Good tolerance
Galla et al. (2017) (112)	65	I,II	RGBM	Bevacizumab 2-15 mg/kg	S	Mannitol 25% 1.4 M	41 patients survived less than 1 year 24 patients survived more than 1 year	–
Faltings et al. (2019) (113)	1	Case report	RGBM	Bevacizumab (15 mg/kg	S	Mannitol 20% 12.5 mL	OS was 24.1 months. Patient with recurrent GBM who had received treatment from 3 clinical trials, including a rechallenge with SIACI of bevacizumab. After the third trial, the MRI scan demonstrated improvement based on Response Assessment In Neuro-Oncology criteria.	Tolerable side effects
Patel et al. (2021) (114)	23	I,II	NGBM	Bevacizumab (15 mg/kg)	S	Mannitol ​​20% (12.5 ml over 120 s)	Median PFS was 11.5 months Median overall survival was 23.1 months	Tolerable side effects
McCrea et al. (2021) (115)	13	I	GBM, DIPG	Bevacizumab (15 mg/kg) with cetuximab (200 mg/m2)	S	12.5 ml of 20% mannitol	The mean overall survival for the 10 DIPG patients treated was 519 days. The ranges for overall survival for the 3 non-DIPG patients were 311–914 days.	epistaxis (2 patients) and grade I rash (2 patients)

GBM, glioblastoma multiforme; NGBM, newly diagnosed glioblastoma multiforme; RGBM, recurrent glioblastoma multiforme;BSG, brain stem glioma; AO, anaplastic oligodendroglioma; O, oligodendroglioma;AAS, anaplastic astrocytoma; AS, astrocytoma; OA, oligoastrocytoma; AOA, anaplastic oligoastrocytoma;MET, metastasis; PCNSL, primary CNS lymphoma; PNET, primitive neuroepithelial tumor; GCT, germ cell tumor; ME, malignant ependymoma; US, unspecified; S, selective;nS, not selective; SD, stable diseases; PR, partial response; PFS, progression free survival; † comparison of intra-arterial/intravenous delivery; SC, single center; MC, multi-center; IA, intra-arterial; IV,intravenous; CR, complete response; DIPG, diffuse intrinsic pontine glioma.

Mannitol continues to be the prevalent BBBD agent, although some recent studies relied on the bradykinin B2 receptor agonist Cereport. As of now (https://clinicaltrials.gov last accessed on 1st of May), there are four clinical trials that have been recently completed (NCT01180816, NCT01238237, NCT00968240, NCT00870181), six are still recruiting (NCT01269853, NCT05271240, NCT02285959, NCT02861898, NCT01884740, NCT02800486), one is active but not recruiting (NCT01811498), one has been suspended (NCT01386710), and one is of an unknown status (NCT03672721). Results available from these trials have been described in detail in the following parts of the review. **Table 2** presents detailed information on ongoing clinical trials relying on IA delivery for the treatment of glioblastoma multiforme and other brain tumours.

**Table 2:** Current clinical trials concerning the use of IA for the treatment of GBM.

Study title	Location	Status	Brain tumour	Estimated enrollment	Method of delivery	Chemotherapeutic, dose	Mannitol dose	Phase
Repeated Super-selective Intraarterial Cerebral Infusion Of Bevacizumab Plus Carboplatin For Treatment Of Relapsed/Refractory GBM And Anaplastic Astrocytoma	Lenox Hill Brain Tumor Center	Suspended	Glioblastoma Multiforme Anaplastic Astrocytoma	54	SSIACI	Bevacizumab Up to 15 mg/kg Carboplatin 150 mg/m^2^	NS	1 2
Super-Selective Intraarterial Cerebral Infusion Of Temozolomide (Temodar) For Treatment Of Newly Diagnosed GBM And AA	Lenox Hill Brain Tumor Center	Completed	Glioblastoma Multiforme Anaplastic Astrocytoma	21	SSIACI	Temozolomide 75–250 mg/m^2^	NS	1
Repeated Super-selective Intraarterial Cerebral Infusion of Bevacizumab (Avastin) for Treatment of Relapsed GBM and AA	Lenox Hill Brain Tumor Center	Recruiting	Glioblastoma Multiforme Anaplastic Astrocytoma	54	SSIACI	Bevacizumab 15 mg/kg	20% 12.5 mL/s	1 2
Super-Selective Intraarterial Intracranial Infusion of Avastin (Bevacizumab)	Lenox Hill Brain Tumor Center	Completed	Glioblastoma Multiforme Anaplastic Astrocytoma	30	SSIACI	Bevacizumab 2–10 mg/kg	NS	1
Super-Selective Intraarterial Cerebral Infusion of Cetuximab (Erbitux) for Treatment of Relapsed/Refractory GBM and AA	Lenox Hill Brain Tumor Center	Completed	Glioblastoma Multiforme Anaplastic Astrocytoma	15	SSIACI	Cetuximab 100–500 mg/m^2^	25% 3–10 mL	1
Super Selective Intra-arterial Repeated Infusion of Cetuximab (Erbitux) With Reirradiation for Treatment of Relapsed/Refractory GBM, AA, and AOA	Lenox Hill Brain Tumor Center	Recruiting	Glioblastoma Multiforme Anaplastic Astrocytoma Anaplastic Oligoastrocytoma	37	SSIACI	Cetuximab 250 mg/m^2^	20% 12,5 mL	2
Super-Selective Intraarterial Intracranial Infusion of Bevacizumab (Avastin) for Glioblastoma Multiforme	Global Neurosciences Institute	Recruiting	Glioblastoma Multiforme	30	SSIACI	Bevacizumab 15 mg/kg	NS	1
Repeated Super-Selective Intraarterial Cerebral Infusion of Bevacizumab (Avastin) for Treatment of Newly Diagnosed GBM	Lenox Hill Brain Tumor Center	Active, not recruiting	Glioblastoma Multiforme Anaplastic Astrocytoma	25	SSIACI	Temozolomide 75–250 mg/m^2^	NS	1 2
IA Carboplatin + Radiotherapy in Relapsing GBM	Université de Sherbrooke	Unknown	Glioblastoma Multiforme	35	IA	Carboplatin 400 mg/m^2^	NA	1 2
Super-selective Intra-arterial Repeated Infusion of Cetuximab for the Treatment of Newly Diagnosed Glioblastoma	Lenox Hill Brain Tumor Center	Recruiting	Glioblastoma Multiforme	33	SSIACI	Cetuximab 250 mg/m^2^	20% 12.5 mL	1 2
ADV-TK Improves Outcome of Recurrent High-Grade Glioma (HGG-01)	Huazhong University of Science and Technology	Completed	Glioblastoma Multiforme	47	IA	Replication-deficient adenovirus mutant ADV-TK, a total of 1 × 10^12^ viral administered in the clinical trial	25% 1.4 M mannitol	2
Oncolytic Adenovirus DNX-2401 in Treating Patients With Recurrent High-Grade Glioma	M.D. Anderson Cancer Center	Recruiting	Anaplastic Astrocytoma Glioblastoma Multiforme Recurrent Gliosarcoma Recurrent Malignant Glioma	36	IA	Oncolytic Adenovirus Ad5-DNX-2401, dose not stated	NS	1
Intraarterial Infusion Of Erbitux and Bevacizumab For Relapsed/Refractory Intracranial Glioma In Patients Under 22	Weill Medical College of Cornell University	Recruiting	Glioblastoma Multiforme Anaplastic Astrocytoma Diffuse Intrinsic Pontine Glioma	30	SSIACI	Erbitux 200 m/m^2^ Bevacizumab 15 mg/kg	Mannitol 25% 10 mL	1 2

NS, not specified; SIACI, super-selective intra arterial cerebral infusion.

Clinical IA trials of the last couple of decades tested the efficacy and safety of IA delivery of platinum analogues (cisplatin and carboplatin), methotrexate, vincristine, nitrosourea derivatives, including carmustine (BCNU), nimustine (ACNU) or 1-(2-hydroxyethyl) chloroethyl nitrosourea (HeCNU), diaziquone, etoposide, and idarubicin. The most recent clinical trials have focused on evaluating the role of new antibodies like bevacizumab and cetuximab. Enrolled patients had surgery and were in favourable clinical condition (10). Although certain studies included patients who had a Karnofsky performance scale score (KPS) of 20, the prevailing majority of clinical trials required a KPS of a minimum of 60 (10).

### Nitrosourea derivatives

First clinical studies of IA nitrosourea derivatives showed encouraging results, but the resulting neurotoxicity quickly diminished the enthusiasm (10, 116–118). In 1986, Feun and colleagues demonstrated in a follow-up phase II trial that IA BCNU may lead to severe leukoencephalopathy and blindness (119). These suggestions were proven valid by Tonn et al. and Kleinschmidt-DeMasters et al., who demonstrated in treated patients a significant risk of local cerebral necrosis as well as leukoencephalopathy (116, 120, 121). Follow-ups of patients have shown that IA BNCU may result in leukoencephalopathy, blindness and increased risk of cerebral necrosis (10, 116, 118–121). The interest in IA nitrosourea derivatives began to wane after a randomised phase III trial comparing IA with IV BCNU showed that IA BCNU is unsafe and lacks effectiveness in regard to increasing patient survival (121). More recently, Fauchon et al. evaluated the role of intracarotid HeCNU (120 mg/m2) in 40 patients before the start of irradiation (122). The authors reported a median TTP of 32 weeks, with an overall median survival of 48 weeks. Neurological toxicity involved visual loss (15%) and leukoencephalopathy (10%) (122).

### Platinum analogs

Ever since Follézou et al. demonstrated that IA of 400 mg/m2 carboplatin led to a partial response in malignant glioma patients, numerous studies evaluating the role of IA of platinum analogs followed (10, 123). Gobin et al. reported the IA delivery of up to 1400 mg/hemisphere of carboplatin in their dose-escalation study based on cerebral blood flow, reporting median survival of 39 weeks and a response rate of 70% (50% SD and 20% PR) of 19 patients (124). In a more recent study, Cloughesy et al. reported a median survival of 11 months (from the time of beginning IA treatment) (111). The regimen involved IA delivery of carboplatin conducted every four weeks for up to 12 cycles (111). Reported toxicity was manageable, with 8% of patients demonstrating grade II neutropenia, 12% of grade II thrombocytopenia and 7% of grade III thrombocytopenia. In summary, the potential for visual loss seems to be greater for patients undergoing IA carmustine and other nitrosoureas than for patients receiving cisplatin or carboplatin.

### Diaziquone, etoposide and idarubicin

Other drugs tested for single-agent IA chemotherapy of recurrent gliomas are diaziquone, etoposide and idarubicin. Greenberg et al. have studied IA diaziquone (10–30mg/m2) in 20 patients with recurrent astrocytomas (125). Two of 20 patients demonstrated partial responses of 5 and 8+ months, respectively. Four patients showed disease stabilisation of 3, 4, 5, and 8 months duration, respectively, and one of these patients achieved tumour shrinkage (125). The reported toxicity was similar to carmustine and cisplatin (125). According to the authors, IA diaziquone was no more effective when using the intravenous approach (125). Intracarotid etoposide (100–650 mg/m2) in 15 patients suffering from recurrent high-grade primary brain tumours demonstrated ambiguous results as some patients had a low objective response rate (7%), while another 33% showed stabilisation of disease over 8–40 weeks (126). More recently, Chehimi et al. have evaluated the efficacy of IA idarubicin (12mg/m2) in two recurrent and progressive GBM patients that had failed after temozolomide and bevacizumab treatment (127). Prior to starting the treatment, the authors tested idarubicin against four human GBM cell lines and observed sensitivity to concentrations in the range of 3 μg/mL of idarubicin (127). On the 3rd day after IA administration, the first patient experienced a neurological event that involved worsening left hemiparesis and severe cognitive impairment, making additional treatment impossible. In contrast, the second patient tolerated IA idarubicin, showing adverse events and a stable follow-up on an MRI scan after 4 weeks (127).

Although the Stupp protocol remains a gold standard for the treatment of GBM since its publication, the idea of IA delivery of temozolomide (TMZ) has been abandoned once studies have reported toxicities and decided that TMZ in its current formulation is unsafe for IA infusion (11, 128). The low efficacy of IA delivery of temozolomide is attributable to the fact that glioblastoma stem cells (GSCs) were proven to be resistant to it (129). In comparison, IA delivery of platinum analogs was associated with a smaller amount of cerebral side effects, especially after the incorporation of selective IA infusion (10, 11, 98, 102). In summary, side effects were shown to be reversible or manageable, proving the safety of IA delivery of platinum analogs. More importantly, according to numerous authors, IA delivery of platinum analogs may lead to a modest response rate and increased time to progression (10, 11, 98, 102, 103). Nonetheless, it is difficult to precisely evaluate the efficacy of platinum analogs as they were used in combination with other drugs (10, 11, 98, 102, 103). Consequently, the efficacy of IA delivery of platinum analogs necessitates further examination (10, 11, 103).

Intra-arterial delivery of carboplatin, methotrexate, cyclophosphamide and etoposide resulted in a high degree of tumour response in chemotherapy-sensitive tumours, such as primary central nervous system lymphoma (PCNSL), primitive neuroectodermal tumour (PNET), germ cell tumour and cancer metastasis to the brain (11, 97). According to a large, multi-institutional study of 149 patients with newly diagnosed primary CNS lymphoma, intracarotid or intra-vertebral IA delivery of methotrexate with osmotic BBB disruption led to a 5-year PFS of 31%, 7-year PFS of 25%, and median OS of 14 years in low-risk patients (11, 106). Nonetheless, results of IA delivery of the aforementioned drugs fall short of providing a relevant benefit in glioblastomas patients (11, 97). IA therapy of these drugs is not superior to IV chemotherapy in the treatment of glioblastomas (9). What could explain this phenomenon is glioblastomas’ significant resistance to various anticancer drugs or the fact that some of these drugs have rapid transit through the CNS and, thus, a limited dwell time (9, 11). Last but not least, the inadequate mixing or streaming of the drug solution within the artery may result in variable drug distribution within the brain or the tumour after the intracarotid delivery (9, 11). Besides ensuring a large patient group and adequate follow-up, future clinical trials should approach these factors to allow for precise evaluation of the efficacy of the given agent.

### Monoclonal antibodies in IA for the treatment of GBM

#### Bevacizumab

**Figure 3:**
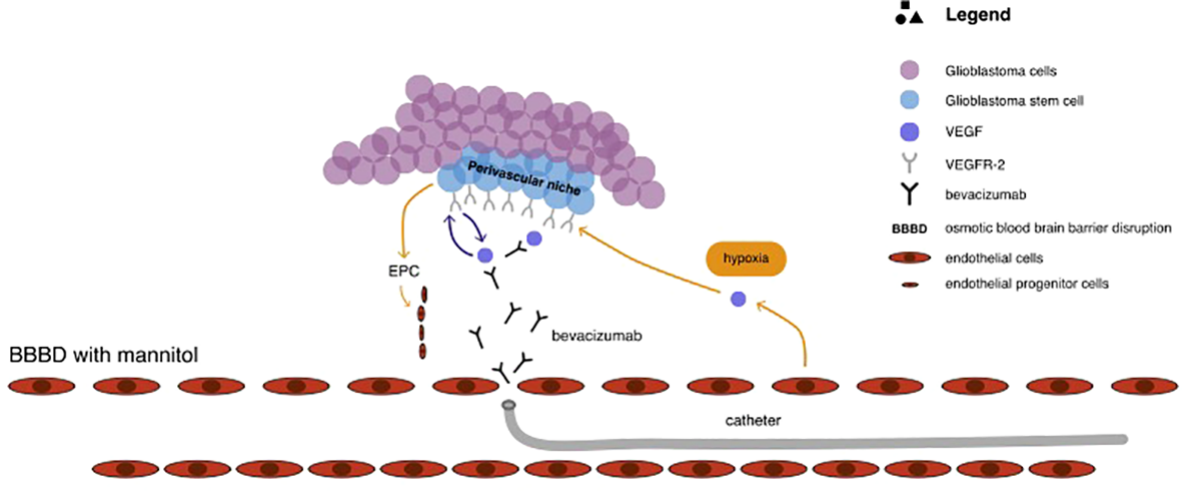
The pharmacological mechanism of catheter delivered bevacizumab in the treatment of glioblastoma.

Vascular endothelial growth factor A (VEGF-A) is the most overexpressed mediator of angiogenesis in glioblastomas multiforme, leading to poorer prognosis (11, 129). This provided rationale for the use of bevacizumab, a monoclonal antibody that blocks the binding of VEGF-A to its receptors in the perivascular niche, which is rich in GSCs and located externally to the luminal side of the vessel (11, 112). The pharmacological mechanism of catheter delivered bevacizumab in the treatment of glioblastoma has been illustrated in **Figure 3**. Results coming from studies, as well as clinical series, have shown that bevacizumab effectively inhibits the formation of new blood vessels and affects the existing brain vasculature leading to vascular normalisation, reduced permeability, and an increase in blood flow velocity (2, 130). This may aid in restoring the normal structure and function of blood vessels as well as decrease tumour-related oedema (130). Although bevacizumab has demonstrated highly encouraging results in patients with newly diagnosed and recurrent GBM by improving 6-month progression-free survival, there are no improvements in terms of overall survival (11, 37, 131, 132). According to Baumgarten, bevacizumab produces different dose-dependent effects on glioma blood vessels and tumour cells (2). Low doses result in a substantial reduction of the total vascular volume without affecting tumour cell viability or the overall tumour growth rates, whereas medium and high doses, besides providing a similar vascular regression, also significantly decrease tumour growth by inhibiting the ability of GSCs to self-renew (2, 10). Furthermore, bevacizumab inhibits the transformation of GSCs into endothelial cell progenitors, which subsequently grow into mature endothelial cells (10, 132). Nonetheless, despite the reasonable response rate during the first few months after bevacizumab treatment, patient survival does not improve as patients still progress and require salvage therapy (10, 108, 133). This may be attributable to the insufficient delivery of bevacizumab through the BBB. Considering that the pore size of BBB is approximately 12nm, bevacizumab, with its size of 15nm, is too big to efficiently penetrate through the BBB (10, 134). Boockvar et al. hypothesised that increasing the concentration of bevacizumab in the perivascular niche could increase the efficacy of inhibiting GCSs, consequently providing better therapeutic results (10). Research focusing on SSIACI of bevacizumab after hyperosmolar BBB disruption for recurrent GBM has evaluated that 15 mg/kg is the maximum tolerated dose (MTD) (10, 11, 108). IA treatment of bevacizumab has an edge over IV treatment, given that studies reported a median PFS of 3.9 months in case of IA bevacizumab and median PFS from 3.3 to 3.7 months in case of IV treatment (11, 135, 136). Chakraborty et al. have evaluated that a single SIACI of BV at 15mg/kg after BBD with mannitol allowed obtaining similar or better PFS in comparison to a biweekly IV infusion of bevacizumab at 10mg/kg (137). Zawadzki et al. have performed three intra-arterial deliveries of bevacizumab under real-time MRI guidance in a patient with butterfly-shaped recurrent glioblastoma as a sole treatment (138). The patient managed to survive 6 months after MRI detection of aggressive regrowth (138). All administrations were safe and uneventful. According to the authors, the therapeutic effects of intra-arterial bevacizumab offered reproducible symptomatic relief which lasted 7-8weeks (138).

It remains unclear if repeated SIACI of bevacizumab after BBBD with mannitol have a long term therapeutic effect, but ongoing clinical trials (**Table 2**) should provide answers (11).

#### Cetuximab

Epidermal growth factor receptor (EGFR) is a potent oncogene, frequently amplified and mutated in high-grade gliomas, prompting diagnosis to be unfavourable (11, 139). Cetuximab is a chimeric human monoclonal antibody that binds and competitively inhibits EGFR, thus reducing signal transduction. Tumour growth is inhibited, and the ultimate result is cell death (81, 139). Given that cetuximab diminishes angiogenesis, combined therapy of cetuximab with bevacizumab could have a synergistic effect on angiogenesis (140). Studies have demonstrated that cetuximab increases activity with radiotherapy and chemotherapy and is capable of mediating antibody-dependent cell-mediated cytotoxicity (81, 140). Phase I study of SIACI of cetuximab after BBBD with mannitol in patients with malignant glioma has estimated that MTD of 250 mg/m is safe and well-tolerated (64). Reported complications included tolerable rash (2 patients), anaphylaxis (1 patient), isolated seizure (1 patient) and seizure with cerebral edema (1 patient) (81). There is an ongoing phase II study aiming at estimating the efficacy of repeated infusion of cetuximab with reirradiation in patients with relapsed/refractory glioblastoma (NCT02800486).

Recently, a phase I trial of 13 paediatric patients with refractory diffuse intrinsic pontine glioma (DIPG) and glioblastoma has shown that super-selective intra-arterial cerebral infusion (SIACI) of bevacizumab (15 mg/kg) and cetuximab (200 mg/m2) is well-tolerated (115). The mean overall survival for the 10 DIPG patients treated was 519 days, whereas the ranges for overall survival for the 3 non-DIPG patients were 311–914 days (115).

### Emerging potential therapeutic approaches

Researchers have been actively evaluating and looking for potential therapeutic agents that could increase the survival of glioblastoma patients. Greenberg et al. have shown that catheter injection of anaerobic radiosensitizer such as bromodeoxyuridine into the external carotid artery led not only to an increased susceptibility of glioma cells to radiotherapy but also to increased survival time of GBM patients (141). Subsequently, research on animal models demonstrated that intra-carotid injection of recombinant human TNF and lymphotoxin allows producing significant anti-tumour effects in C6 and T9 gliomas (142). Yoshida et al. reported a 20% response rate after a non-selective administration of recombinant human tumour necrosis factor-a in malignant glioma patients (143).

Tumour-treating fields (TTFields) have been hypothesised as yet another potential treatment for recurrent as well as newly diagnosed glioblastoma. By delivering low-intensity (1-3 V/cm), intermediate-frequency (100-300 kHz) alternating electric fields *via* transducer arrays applied to the scalp, TTFields lead to mitotic arrest and apoptosis of quickly dividing cells. A randomised Phase III clinical trial involving 237 patients with recurrent glioblastoma, in whom prior therapy had failed, compared the TTFields as a monotherapy to chemotherapy (144). Even though there was no statistically significant difference in regard to survival, TTFields demonstrated efficacy and activity similar to the chemotherapy regimens, with lesser toxicity and overall improvement in quality of life (144). A 2009 Phase 3 clinical trial involving patients with newly diagnosed glioblastoma found that adding TTFields to maintenance temozolomide chemotherapy resulted in statistically significant improvement in survival (6.7 months vs 4.0 months) (145)

#### Gene therapy

Animal studies have shown the potential therapeutic benefits associated with gene therapy. The growth of Gli36 glioblastoma tissue carrying a missense-mutated p53 gene can be impeded by intra-arterial delivery of a p53-containing adenoviral vector, whereas intra-arterial administration of a plasmid encoding anti-angiogenic endostatin resulted in decreasing tumour vascular density, perfusion, and permeability, consequently allowing to prolong survival time in the rat 9L gliosarcoma model (69, 146, 147). A Phase II clinical trial evaluated an intra-arterial delivery of ganciclovir combined with replication-deficient adenovirus mutant thymidine kinase. Results demonstrated a significant improvement in 6-month progression-free survival, overall progression-free survival, and overall survival in patients suffering from recurrent high-grade gliomas (69, 148).

#### Cell therapy

A concerted effort in the development of new glioblastoma treatments has led to animal studies evaluating the role of cell therapy (69). Goerger and colleagues have shown that early-stage intracarotid delivery of a human cytotoxic T-cell line (TALL-104) in the 9L glioblastoma model significantly increased survival rates (149). A study on animal models has shown that injection of a murine colon cancer cell line (CT-26) overexpressing interleukin-4 (IL- 4) or hemagglutinin antigen resulted in systemic immunity against liver and lung metastases but not against brain metastases (150).

Recently, the interest has shifted towards genetic engineering of T cells to express chimeric antigen receptors (CARs) directed against specific antigens (151). Once the tumour-associated antigen is identified, CAR T cells specific to that antigen can induce antitumor responses in a human leukocyte antigen (HLA)-independent manner (151). Early results of systemic delivery have shown safety and optimistic results in regard to efficacy (151). However, intra-arterial delivery has been only evaluated in studies with liver metastases due to colorectal cancer, demonstrating safety and encouraging results (152, 153). Nonetheless, the possibility of any therapeutic applications is strongly limited by the scarcity of research and lack of clinical trials.

### What lays ahead

As much as the renewed interest brought new advancements and progress with IA therapies to neuro-oncology, there is still massive room for improvement. Glioblastomas multiforme constitute a highly heterogeneous entity, both functional and morphologic (129, 154). Although *in vitro* all clones demonstrate neuronal precursor phenotype, individual clone-derived populations overexpress various different GBM markers (such as EGFR, EGFRvIII, and PTEN) and characterise by a dissimilar response to a variety of drugs (129). Considering this, the likelihood of a single therapeutic agent which will be effective for the treatment of glioblastoma multiforme is extremely low. Thus, it is paramount to establish an adequate multimodal therapy, which will have a synergistic effect on the diverse pathogenesis of GBM (11, 129). Other approaches include a personalised choice of intra-arterial therapy based on tumour genetic phenotype and *in-vitro* testing. There are multiple mechanisms underlying the drug resistance of glioblastomas, depending on both tumour-intrinsic factors and tumour microenvironment-dependent factors. Effective treatment for glioblastoma demands obtaining detailed pathological, genomic, transcriptomic, and epigenetic data to precisely determine the source of drug resistance (7). Considering that there are numerous mechanisms of resistance and the high intra-tumour heterogeneity of glioblastoma, precision medicine will undoubtedly have to rely on multiple drugs leading to a synergistic effect (7).

As much as hyperosmolar disruption of the BBB is an effective and popular technique, a more profound understanding of the pharmacological kinetics of BBB will allow estimating the most effective dose for a specific agent (11, 29). In order to obtain the best results possible with SIACI, technical aspects such as the selection of cerebral vessels, incorporation of catheters with balloons, flow arrest, or pulsatile injections have to be adequately incorporated (11). Recent preclinical and clinical studies have demonstrated that adding MRI to guide IA infusion rather than relying solely on X-ray is highly advantageous (155, 156). Considering the low sensitivity of contrast agents, angiography demands a rapid bolus infusion of contrast, which significantly limits the visualisation of the smallest intracranial vessels (156). However, MRI contrast agents have a high sensitivity that allows detection of the smallest concentrations of contrast, particularly at the microcirculation level (156). MRI guidance provides the unique possibility of showing the territory of the brain parenchyma supplied by the catheter, which tends to be extremely dynamic and variable. Furthermore, MRI guidance permits modification of the infusion rate and catheter tip so that the infusion can be limited to the targeted.

**Figure 4:**
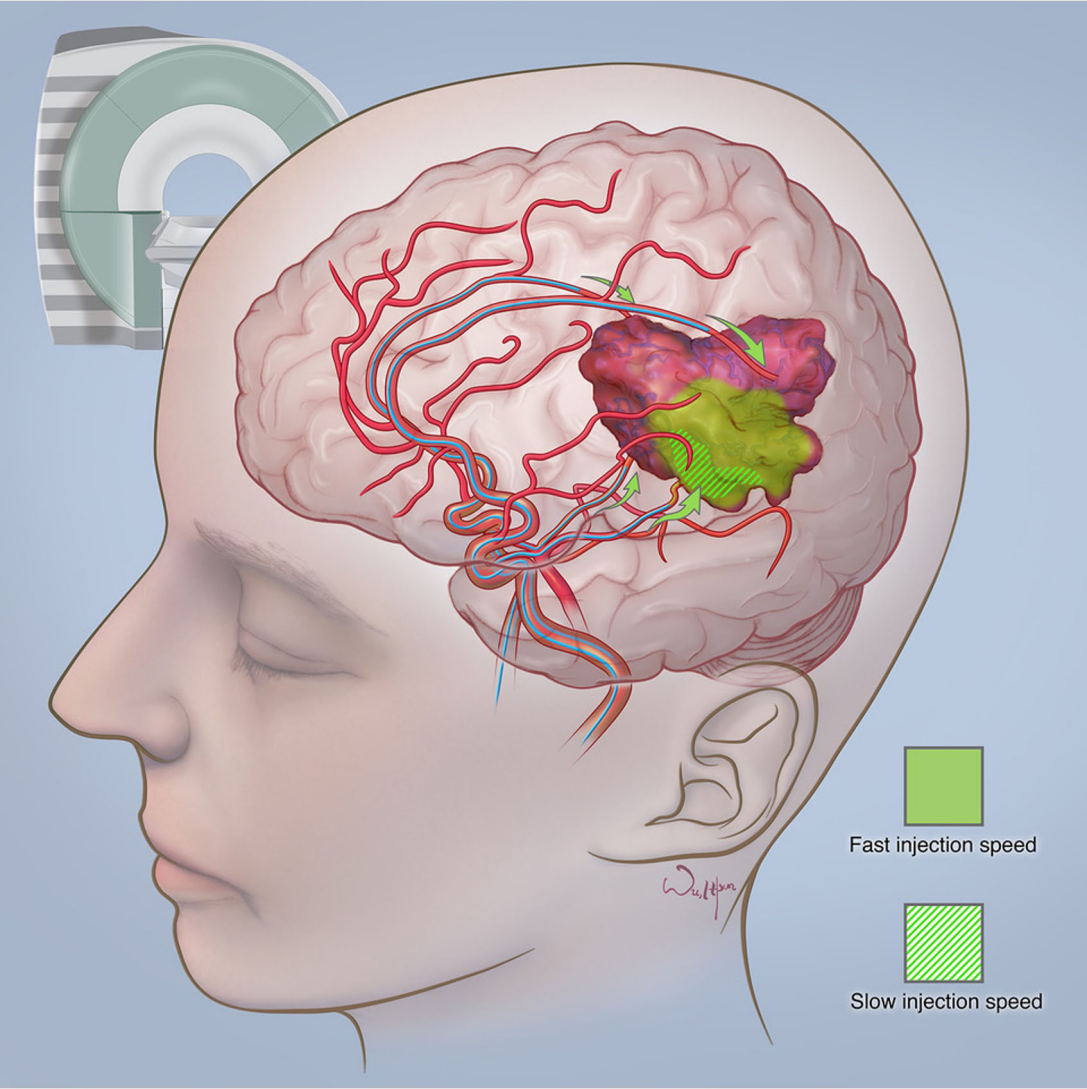
The difference between fast and slow infusion rates and their influence on drug delivery. Courtesy of the Society of Image guided Neurointerventions (SIGN).

Zawadzki et al. have reported the first-in-man targeted intra-arterial cerebral infusion under real-time MRI guidance to be technically feasible and safe (156). Real-time MRI guidance during microcatheter infusions offered essential quantification of the degree of overlap between the transcatheter perfusion territory and the enhancing mass, greatly helping in the selection of the faster infusion rate (156). The difference between fast and slow infusion rates and their influence on drug delivery has been illustrated in **Figure 4**. Given the variable vascularity of glioblastoma, angiography may fall short of localising the exact vascular supply of GBM (156, 157). According to Chen et al., who reported the first use of perfusion guidance during the infusion of mesenchymal stem cells loaded with Delta-24 (MSC-24) in the treatment of glioblastoma, the combination of preoperative anatomic MR images with real-time perfusion images from super-selective injection during angiography allows for accurate identification the vascular supply, consequently facilitating more effective intra-arterial delivery of chemotherapeutics (157). Cone-beam computed tomography (CBCT), being an inherent part of planning IA injection and determining the area of infusion, allows for generating perfusion maps, which greatly optimise the accuracy of IA delivery, limiting exposition of healthy brain parenchyma to delivered chemotherapeutics (157). Furthermore, uncomplicated determination of the perfusion volume facilitates the calculation of the adequate dose. However, what is still a limitation of this technique is the high dose of radiation during each cone-beam CT acquisition and the lack of real-time visualisation of administered drug distribution (157).

Impressive advancements in artificial intelligence throughout the last decade have resulted in the use of deep learning approaches, known as convolutional neural networks (CNNs), in glioma patients (158). Besides using MR data to grade gliomas and predict overall survival, different CNNs are used to predict the genetics of glioma on pre-operative MR images. According to a recent review, CNNs are effective in tumour grading and prediction of IDH mutation, 1p19q codeletion, MGMT promoter status, and OS, with accuracies of prediction reaching 80% to 90% (158).

There is a need for new robust pharmacokinetic models which will take into consideration hydrodynamic factors. It has been established that hydrodynamic factors such as the background blood flow, injection characteristics and vascular geometry have a significant role in determining tissue concentrations after IA drug injections (86). The advent of nanotechnology should also be taken into consideration, as smaller particles are subjected to substantially smaller hydrodynamic forces (86). Real-time tracking of tissue drug distribution and concentrations such as PET could significantly help in establishing reliable models (11, 86) Likewise, real-time monitoring of BBB disruption is essential for the improvement of IA cerebral infusions of chemotherapeutics in the treatment of glioblastoma multiforme. According to Kiviniemi et al., direct-current electroencephalography (DC-EEG) can be used to monitor the induced transient BBBD in anaesthetized human patients undergoing chemotherapy for PCNSL (13). DC-EEG allows for characterization of the spatiotemporal behaviour of scalp-recorded slow electrical signals during blood-brain barrier opening (13). The authors also monitored the patients with near-infrared spectroscopy (NIRS) in order to obtain information on cerebral hemodynamics that has a role in DC-EEG signal generation (13). Future clinical trials using IA delivery of chemotherapeutics in glioblastoma patients should try evaluating the use of DC-EEG for real-time monitoring of BBBD.

## Conclusions

It is widely recognized that the intra-arterial route of administration ensures higher drug concentrations in targeted areas, limits systemic toxicity and is safe in experienced hands. However, although the results coming from various phase I studies are promising, due to the lack of phase III clinical trials, with only single-phase 1/phase 2 study reporting outcomes so far, it is impossible to declare the efficacy of IA delivery of chemotherapeutics in the treatment of glioblastoma multiforme. There are numerous areas of improvement necessary for the optimization of this technique and the treatment of GMB. These include: establishing an adequate multimodal therapy, which will have a synergistic effect on the diverse pathogenesis of GBM; relying on the combination of preoperative anatomic MR images with real-time perfusion images from super-selective injection during angiography to accurately identify the vascular supply; conducting precise quantitative and spatial monitoring necessary to guarantee the accurate delivery of the therapeutic to the tumour and estimating the most effective dose of a specific agent for hyperosmolar BBB disruption. Considering the significant heterogeneity of GBM, treatment should be individualised to each patient after obtaining detailed pathological, genomic, transcriptomic, and epigenetic data. Quantum leaps in intrathecal, intracavitary and convection-enhanced delivery, or pharmacological advancements leading to the development of nanoparticles capable of effectively passing BBB, all could potentially challenge the whole premise of intra-arterial delivery. Nonetheless, we believe that the idea of IA infusion for the treatment of malignant brain tumours guided by the fusion of pre-procedural brain MRI to intra-procedural CBCT will not be abandoned for the sake of other methods of drug delivery. It is because controlled and highly precise catheter infusions are not only extremely effective at ensuring high local concentrations of the chemotherapeutic but are safe in experienced hands. With the development of effective agents against glioblastoma, intra-arterial cerebral infusions have the potential to become the mainstay of glioblastoma treatment and offer patients a chance at longer survival.

## Data availability statement

The original contributions presented in the study are included in the article/supplementary material. Further inquiries can be directed to the corresponding author.

## Author contributions

MaP analysed data and drafted the initial form of the manuscript. MiP analysed data, drafted the manuscript and created tables. JW edited the manuscript and analysed data. MZ analysed data, edited the manuscript and approved its final form. All authors contributed to the article and approved the submitted version.

## Conflict of interest

The authors declare that the research was conducted in the absence of any commercial or financial relationships that could be construed as a potential conflict of interest.

## Publisher’s note

All claims expressed in this article are solely those of the authors and do not necessarily represent those of their affiliated organizations, or those of the publisher, the editors and the reviewers. Any product that may be evaluated in this article, or claim that may be made by its manufacturer, is not guaranteed or endorsed by the publisher.
